# Association between Hepatitis B Virus Infection and Metabolic Syndrome in Southwest China: A Cross-sectional Study

**DOI:** 10.1038/s41598-020-62609-4

**Published:** 2020-04-21

**Authors:** Li-Bo Yan, Juan Liao, Ning Han, Ling-Yun Zhou, Xue-Er Wang, You-Juan Wang, Hong Tang

**Affiliations:** 1 Center of Infectious Diseases, West China Hospital of Sichuan University, Chengdu, China; 2 Department of Forensic Pathology, Medical School of Basic and Forensic Sciences Sichuan University, Chengdu, China; 3 Health Management Center, West China Hospital of Sichuan University, Chengdu, China

**Keywords:** Endocrine system and metabolic diseases, Hepatitis, Liver diseases

## Abstract

The correlation between hepatitis B virus (HBV) infection and metabolic syndrome (MetS) remains to be clarified. In this study, we explored this association in a large population in Southwest China. This was a cross-sectional study, with pooled adult health data. Multivariate logistic regression analysis, controlling for age, sex, HBV status, alanine aminotransferase, and fatty liver, was used to identify predictor(s) of MetS. Of the 96,175 participants, positive HBV was identified in 7984 (8.30%) and MetS in 12,092 (12.57%). The MetS prevalence was lower among HBV positive than negative individuals (11.64% *versus* 12.66%, P < 0.001). The adjusted odds (aOR) of positive HBV among individuals with MetS was 0.841 (95% confidence interval (CI), 0.771–0.916) in men and 0.834 (95% CI, 0.672–0.925) in women. Elevated triglyceride level, a component of MetS, was inversely associated with HBV status in both men and women: aOR, 0.551 (95% CI, 0.514–0.590) and 0.683 (95% CI, 0.605–0.769), respectively. Among HBV positive individuals, liver cirrhosis was more common among those with than without MetS (4.83% *versus* 2.93%, respectively; P = 0.002). HBsAg-seropositive are inversely associated with MetS, especially elevated triglycerides. Liver cirrhosis was more common among HBV infection patients with MetS.

## Introduction

Chronic hepatitis B virus (HBV) infection is a major public health issue^1^. HBV infection is not only the cause of acute and chronic hepatitis, but is also one of the key etiological factors of liver cirrhosis and hepatocellular carcinoma (HCC). In 2015, it was estimated that 257 million people, 3.5% of the world’s population, were living with a chronic HBV infection^2^. In China, a HBV-endemic region, the prevalence of HBV infection for population among individual 1 to 59 years of age is approximately 7.18%, with an estimated 93 million patients individuals in China living with a chronic HBV infection^3^. Although the health morbidities of chronic HBV infection are decreasing, these remain a tremendous healthcare burden in China. Considering that the liver plays a key role in glucose homeostasis and lipid metabolism, the possible association between liver disease and diabetes mellitus or metabolic syndrome (MetS) has been a topic of healthcare research interest.

MetS refers to diseases caused by metabolic disturbances, such as increased waist circumference, hyperglycemia, elevated blood pressure (BP), and dyslipidemia. MetS affects approximately one-fifth in China, reflecting the increasing prevalence of obesity^4,5^. MetS is characterized by dyslipidemia and glucose metabolism disorders, both of which are influenced by hepatic function. In fact, an association between MetS and non-alcoholic fatty liver disease (NAFLD), chronic hepatitis C (CHC) and HCC has previously been demonstrated^6,7^. Additionally, NAFLD is considered as the hepatic manifestation of MetS^8^. It is also now widely confirmed that chronic hepatitis C virus infection may increase the risk of insulin resistance and type 2 diabetes^9^. MetS is also a possible risk factor for HCC, independent of the hepatitis virus status.

The relationship between HBV infection and MetS has been explored in several studies^10–18^, with six of these studies focusing on Asian populations^12–17^, and two on American populations10,11. Despite this body of research, the correlation between HBV infection and MetS remains unclear^11,19^. Of note, most studies have indicated that the HBV surface antigen(HBsAg) is positivity inversely associated with MetS^11,20^. Moreover, a study performed at a university health center in a North Taiwan province reported that anti-HBc(+) HBV infection was associated with a higher risk for MetS^19^. Our aim in this study was to evaluate the association between HBV infection and MetS, and the individual components of MetS, in China, where the public health burden of HBV infection is high, with an estimated 93 million cases of HBV infection and an endemic rise in MetS.

## Results

### Baseline characteristics

Our study cohort included 96175 participants, of which 7984 (8.30%) were HBsAg seropositive, and the other 88191(91.70%) HBsAg seronegative. Basic demographics and characteristics of participants with and without HBV infection are shown in Table 1. When compared to the non-infection group, the chronic HBV group had a higher proportion of males (62.84% versus 53.59%, respectively, P < 0.001), were older (mean age, 45.1 ± 10.9 years versus 44.8 ± 12.3 years, respectively, P = 0.0154), and had a lower urban population (78.31% versus 83.72%, respectively, P < 0.001). ALT levels were higher among individuals with than without HBV infection (26(19–38) IU/L versus 21(15–32) IU/L, P < 0.001). As well, the BMI and waist circumference measurements were higher among individuals with than without HBV infection, while levels of total cholesterol, HDL cholesterol, and triglyceride were lower (P < 0.05). Fasting glucose, systolic pressure and diastolic blood pressure were similar between these two group (P > 0.05). MetS was identified in 12.57% of the study sample (n = 12092/96175), with a lower proportion among adults with (11.64%) than without (12.66%) HBV infection (P < 0.001). The proportion of individuals with fatty liver was lower among individuals with than without HBV (21.05% versus 23.84%, P < 0.001).

**Table 1:** Baseline characteristics of all participants.

	Total (n = 96175)	HBV infection (n = 7984)	Non-HBV infection (n = 88191)	P
Age	44.8 ± 12.1	45.1 ± 10.9	44.8 ± 12.3	0.0154
Gender(male), n(%)	52283 (54.36%)	5017 (62.84%)	47266 (53.59%)	<0.001
Location(City), n(%)	80095 (83.28%)	6253 (78.31%)	73842 (83.72%)	<0.001
BMI(kg/m2)	23.6 ± 3.26	23.9 ± 3.17	23.6 ± 3.26	<0.001
Systolic pressure (mm Hg)	118 ± 16.2	117 ± 15.8	117 ± 16.8	0.254
Diastolic pressure (mm Hg)	73.7 ± 10.6	73.9 ± 10.7	73.7 ± 10.6	0.158
Waist circumference (cm)	80.4 ± 10.2	81.7 ± 10.0	80.3 ± 10.2	<0.001
waist to hip ratio	0.85 ± 0.07	0.86 ± 0.07	0.85 ± 0.08	<0.001
ALT (IU/L)*	22 (15–32)	26(19–38)	21 (15–32)	<0.001
AST (IU/L)*	23 (19–28)	25(21–32)	23 (19–28)	<0.001
glucose (mmol/L)	5.20 ± 1.15	5.18 ± 1.19	5.20 ± 1.15	0.13
Total cholesterol (mmol/L)	4.88 ± 0.933	4.70 ± 0.881	4.89 ± 0.936	<0.001
HDL cholesterol (mmol/L)	1.45 ± 0.402	1.43 ± 0.402	1.45 ± 0.402	<0.001
LDL cholesterol (mmol/L)	2.80 ± 0.77	2.69 ± 0.72	2.81 ± 0.77	<0.001
Triglyceride (mmol/L)	1.57 ± 1.28	1.41 ± 1.08	1.58 ± 1.30	<0.001
Metabolic syndrome n(%)	12092 (12.57%)	930 (11.64%)	11162 (12.66%)	<0.001
Elevated triglyceride n(%)	28167 (29.29%)	1801 (22.56%)	26366 (29.89%)	<0.001
Elevated blood pressure n(%)	24681 (25.66%)	2014 (25.22%)	22667 (25.70%)	0.2835
Elevated blood glucose n(%)	7905 (8.21%)	660 (8.27%)	7245 (8.21%)	0.9019
Low HDL cholesterol n(%)	14057 (14.61%)	1254 (15.70%)	12803 (14.51%)	0.0041
Abdominal obesity n(%)	21026 (21.86%)	1957 (24.51%)	19069 (21.62%)	<0.001
Fatty liver n(%)	22798 (23.70%)	1681 (21.05%)	21117 (23.84%)	<0.001
HBeAg status				
HBeAg positive n(%)	608 (0.63%)	589 (7.38%)	19 (0.00%)	<0.001
HBeAb positive n(%)	28622 (29.76%)	7269 (91.04%)	21353 (24.21%)	

ALT, alanine aminotransferase; AST, aspartate aminotransferase; HDL-C, high-density lipoprotein cholesterol; LDL-C, low-density lipoprotein cholesterol.

*ALT,AST are non-normally distributed quantitative variables, expressed as median values (interquartile range).

### HBV infection and metabolic syndrome

To control for potential risk factors for MetS between the two groups, we used two regression models to evaluate the association between HBV infection and MetS, with results for the unadjusted and adjusted multivariate logistic regression analyses reported in Table 2. Model 1controlled for age and location, while model 2 controlled for age, location, ALT level, and presence of NAFLD. For model 1, the odds ratio (OR) for seropositive HBsAg among individuals with MetS was 0.783 (95% confidence interval (CI), 0.724–0.847) in men and 0.785 (95% CI, 0.640–0.954) in women. In model 2, the OR for seropositive HBsAg among individuals with MetS was increased to 0.841 (95% CI, 0.771–0.916) in men and 0.834 (95% CI, 0.672–0.925) in women. Overall, a HBsAg-seropositive status was inversely associated with MetS in both men and women.

**Table 2:** Odds ratio of the metabolic syndrome in chronic hepatitis B individuals compared to controls.

Model	Men (n = 52283)			Women (n = 43892)		
metabolic syndrome	OR	95%CI	P	OR	95%CI	P
Unadjusted	0.779	0.720–0.842	<0.001	0.847	0.691–1.017	0.082
Model 1	0.783	0.724–0.847	<0.001	0.785	0.640–0.954	0.017
Model 2	0.841	0.771–0.916	<0.001	0.834	0.672–0.925	0.022

Model 1: Adjusted for age and location.

Model 2: Adjusted for age, location, ALT and Fatty liver.

CI, confidence interval.

### HBV infection and the components of metabolic syndrome

Outcomes of the multivariable analyses, performed to explore the associations between an HBsAg-seropositive status and the five components of MetS, are reported in Table 3. Among individual components of MetS, the adjusted risks (aOR) of having elevated triglyceride was significantly lower in both men and women with an HBsAG-seropositive status(OR, 0.551, 95% CI, 0.514–0.590, and aOR, 0.683, 95% CI, 0.605–0.769, respectively). Of note, the adjusted risk of having hypertension was lower (0.885, 95% CI: 0.828–0.946) among men with an HBsAg seropositive than seronegative status. HBsAg-seropositive status was inversely associated with an elevated triglyceride level in both men and women, and an elevated blood pressure in men.

**Table 3:** Odds ratio of the metabolic abnormalities in chronic hepatitis B individuals compared to controls.

Model	Men			Women		
	OR	95%CI	P	OR	95%CI	P
Elevated triglyceride						
Unadjusted	0.558	0.523–0.595	<0.001	0.732	0.653–0.817	<0.001
Model 1	0.562	0.527–0.599	<0.001	0.691	0.615–0.774	<0.001
Model 2	0.551	0.514–0.590	<0.001	0.683	0.605–0.769	<0.001
Elevated blood pressure						
Unadjusted	0.852	0.800–0.909	<0.001	1.033	0.936–1.137	0.51
Model 1	0.865	0.810–0.924	<0.001	0.963	0.865–1.070	0.49
Model 2	0.885	0.828–0.946	<0.001	0.970	0.871–1.079	0.58
Elevated blood glucose						
Unadjusted	0.842	0.856–1.034	0.214	0.889	0.726–1.064	0.213
Model 1	1.002	0.909–1.103	0.955	0.855	0.704–1.030	0.107
Model 2	1.096	0.991–1.209	0.070	0.879	0.720–1.065	0.199
Low HDL cholesterol						
Unadjusted	0.939	0.875–1.007	0.079	1.077	0.897–1.282	0.414
Model 1	0.934	0.871–1.001	0.057	1.042	0.868–1.242	0.645
Model 2	0.997	0.926–1.072	0.938	1.097	0.910–1.313	0.318
Abdominal obesity						
Unadjusted	1.044	0.980–1.112	0.174	1.091	0.973–1.220	0.131
Model 1	1.048	0.984–1.117	0.140	1.010	0.895–1.137	0.864
Model 2	1.147	0.969–1.230	0.225	1.041	0.916–1.180	0.528

Model 1: Adjusted for age and location.

Model 2: Adjusted for age, location, ALT and Fatty liver. CI, confidence interval.

### Metabolic syndrome and liver cirrhosis in patients with HBV infection

Among individuals with an HBV infection, liver cirrhosis was more common among those with than without MetS (4.83% versus 2.93%, P = 0.002; Table 4). By comparison, among individuals with a negative HBV infection status, the prevalence of liver cirrhosis was similar between those with than without MetS (Table 4). After controlling for age, location, sex, ALT, and fatty liver status, MetS remained an independent risk factor for liver cirrhosis (aOR, 1.85; 95% CI, 1.2–2.7). Therefore, MetS increases the risk for liver cirrhosis among patients with HBV infection.

**Table 4:** Metabolic syndrome increases the risk of liver cirrhosis in patients with HBV infection.

	HBV infection		χ2	P	Non- HBV infection		χ2	P
	Mets (N = 930)	Non-Mets (N = 7054)			Mets (N = 77029)	Non-Mets (N = 11162)		
Non- Cirrhosis (N,%)	885 (95.17%)	6847 (97.07%)	9.75	0.002	76989 (99.94%)	11151 (99.91%)	3.67	0.056
Cirrhosis (N,%)	45 (4.83%)	207 (2.93%)			40 (0.06%)	11 (0.09%)		

χ^2^, chi-squared test.

## Discussion

The prevalence of HBV infection in Southwest China in our study was 8.30%, compared to the prevalence rate of 7.18% for the general population, between the ages of 1 to 59 years, in a nationwide survey in China^3^. Previous studies have reported prevalence rates of HBsAg infection for the age groups of 1–4 year, 5–14 years, and 15–29 years of 0.32%, 0.94%, and 4.38%, respectively^21,22^. Therefore, the prevalence of HBV infection is lower among younger individuals. Our study group included only adults and as such, the prevalence of HBV infection among our study group was slightly higher than the prevalence for the general population.

The relationship between HBV and MetS remains inconclusive^10–18^, with some studies reporting a protective effect of HBsAg seropositivity against MetS while other studies found no association between HBsAg positivity and MetS^20^. It is important to note, however, that most studies regarding the association between HBV and MetS have been performed in Taiwan, Japan, Korea, Slovakia and the United States, with few studies having been conducted in China. Our large-scale cross sectional study reflects the association between HBV infection and MetS in Southwest China, showing an inverse association between HBsAg-seropositivity and MetS in this population. These results are consistent with those of with previous Asian studies^12,13^. In agreement with our findings, Huang et al. reported a lower risk of MetS among individuals with a HBsAg-seropositive than a seronegative status, after controlling for sex and age (OR, 0.76; 95% CI, 0.68–0.85)^12^. Jinjuvadia *et al*. similarly reported an inverse association between HBV infection and MetS in a large population database in the United States^11^. An OR for HBsAg-seropositivity among men with MetS of 0.32 (95% CI. 0.12–0.84) has previously been reported, with a meta-analysis reporting an OR for MetS of 0.80 (95% CI, 0.70–0.90) among individuals with an HBsAg-positive status compared to controls^20^. One study did report a higher risk of MetS with an HBV infection presenting with anti-HBc(+)^19^. This difference may be due to the distinctive definition of hepatitis B infection by anti-HBc(+).

In our study, we found that HBsAg-seropositive participants had lower total cholesterol, HDL cholesterol and triglyceride levels than the seronegative (control) group. After controlling for multiple factors, HBV infection found to be inversely associated with elevated triglycerides in both men and women. There is in fact accumulating evidence of an inverse association between HBV infection status and all lipid profiles including cholesterol, triglyceride, HDL cholesterol and low-density lipoprotein cholesterol (LDL-C)^23^. A study involving 17,030 residents in Taiwan also reported that HBsAg seropositivity was inversely associated with hypertriglyceridemia^12^. In this study, the authors included constructed a structural equation model revealed HBV infection had a significant negative effect on hypertriglyceridemia^12^. Similarly with our study, a large-scale cohort study found that HBV seropositivity was associated with a lower prevalence of both hypertriglyceridemia and hypercholesterolemia^24^. In brief summary, HBV infection has an inverse association with lipid profiles.

In this large-scale cross-sectional study, we found HBsAg-seropositive participants had higher ALT levels than the seronegative (control) group. It indicated that some HBsAg-seropositive participant are with active hepatitis and need antiviral treatment. We found that liver cirrhosis was more common among patients with a chronic HBV infection patients with MetS compared to those chronic HBV infection without MetS. Another study performed in Hong Kong has also clearly shown that MetS is independently associated with liver cirrhosis among individuals with chronic HBV infection (OR 1.7, 95% CI 1.1–2.6)^25^. An prospective cohort study using paired transient elastography examinations also indicated that coincidental MetS increases the risk of progression of liver fibrosis in patients with chronic HBV infection^16^. Lifestyle modifications should be advised to patients with HBV infection at risk of MetS.

The underlying mechanism of the association between HBV infection and MetS is not fully understood. HBV is a hepatotrophic virus that can cause hepatocyte injury. Liver is the center of glucose homeostasis and lipid metabolism. The Na+ taurocholate cotransporting polypeptide (NTCP) was identified as the functional cellular receptor mediating HBV entry. NTCP also responsible for bile acid (BA) uptake by hepatocytes. Oehler et al. found that HBV binding to NTCP promoting compensatory BA synthesis and cholesterol provision^26^. HBV X protein (HBx), encoded by X gene in HBV genome, is a multifunctional regulator that modulates gene transcription, signaling pathways and tumorigenesis. Several studies indicated that HBx causes hepatic steatosis in hepatocyte mediated by transcriptional activation of sterol regulatory element-binding protein 1 (SREBP1) and peroxisome proliferator-activated receptor gamma (PPARγ). Our previous study also demonstrated that HBx related differentially expressed proteins are associated with lipid metabolism^27^. HBx induces abnormal lipid metabolism to meet the bioenergetic demands of extreme cell growth and proliferation^28,29^. Kang *et al*.^30^ reported that HBx inhibits the secretion of apolipoprotein B, an important glycoprotein for the transport of cholesterol in the liver. The association between a lower rate of metabolic syndrome among patients with HBV infection and the increase in some genes associated with lipid and bile acid metabolism might be due to lipid transporter dysfunction in hepatocytes.

The limitations of our study should be acknowledged. Firstly, demographic details including income status, education levels, cigarette smoking, level of physical activity and medications were not recorded during the medical examinations and, therefore, could not be included in our analysis. Secondly, we do not have the data on HBV viral load to further explore the metabolic components between different HBV subgroups, and the prevalence of HBV infection might be underestimated due to occult HBV infection. Thirdly, the participants who had medical examination at our hospital were enrolled in this cross-sectional study. This study is not a large-scale epidemiological study of random sampling. Lastly, this is a cross-sectional study, and therefore it was not possible to explore a cause relationship between HBV infection and MetS. Well-designed prospective observational is need to explore the cause relationship. Pathophysiological studies are also needed to explore the possible biological mechanisms involved in the observed association between HBV infection and MetS.

In conclusion, HBsAg-seropositivity is inversely associated with MetS, especially elevated triglycerides. The inverse relationship between HBV infection and MetS may be attributable to triglycerides. Liver cirrhosis was more common among HBV infection patients with MetS. Well-designed prospective observational is need to explore the cause relationship.

## Patients and Methods

### Study design

We used a cross-sectional design with pooled data from the Physical Examination Center of the West China Hospital of Sichuan University. In all, 96,175 individuals who completed a health examinations (including tests for HBV markers) and lifestyle questionnaires were included from December 2014 to December 2017, were included. We included only individuals who were 18 years of age or older at the time data were collected.

Individuals were excluded if they (1) had missing values for HBsAg serology; (2) had a HCV infection (defined as HCV antibody seropositivity); (3) had missing parameters for individual components of MetS; and/or (4) were pregnant during the data collection period. Informed consent was obtained from all the participants involved in this study. Approval to analyze the demographic and biological data in these subjects was obtained from the Ethic Committee of West China Hospital of Sichuan University. All methods were carried out in accordance with relevant guidelines and regulations.

### Clinical evaluation and laboratory tests

The following demographic data were collected: age, race, gender and address. Anthropometric measurements including body weight, height, waist to hip ratio, and blood pressure were performed by trained nurses. Waist circumference was measured at umbilical level or midway between the iliac crest and the lowest rib. Blood pressure measurements were obtained from the uncovered right upper arm, with individual in a sitting posture and after a rest period of at least 10 min. The body mass index (BMI) was calculated by dividing weight (kg) by the height squared (m^2^).

Venous blood samples were collected after an 8 hours overnight fasting period in the morning. A Hitachi Modular analysis system (Roche Modular DPP, Hitachi Ltd., Tokyo, Japan) was used to measure serum levels of alanine aminotransferase (ALT), aspartate aminotransferase (AST), glucose, total cholesterol, triglycerides and high-density lipoprotein cholesterol (HDL-C). A microparticle enzyme-linked immunosorbent assay (Intec Products Inc., Xia men, China) was used to measure status of HBsAg, HBeAg, and antibody to HBeAg, as per previously reported methods^31^.

### Diagnosis of HBV infection, liver cirrhosis and fatty liver

HBV infection was defined by a positive HBV surface antigen (HBsAg) test. Liver cirrhosis was diagnosed by the observation of liver nodules or splenomegaly on abdominal ultrasound, combined with platelet count below 100 000, and/or liver stiffness values >13 Kpa, measured using a fibroscan. Fatty liver was diagnosed when both bright liver and an augmentation of hepato-renal contrast were observed concurrently by ultrasonography.

### Definition of metabolic syndrome

The definition of MetS was based on the guideline for type 2 diabetes as recommended by Chinese Diabetes Association (CDS 2018). Specifically, MetS was defined by the presence of at least three of the following: (1) a high waist circumstance (≥90 cm in men, ≥85 cm in women); (2) impaired fasting glucose (fasting glucose ≥ 6.1 mmol/l or a medical history of diabetes mellitus); (3) elevated blood pressure (systolic blood pressure ≥ 130 mmHg, or diastolic blood pressure ≥ 85 mmHg, or use anti-hypertensive medications; (4) a triglyceride level (TG) ≥ 1.7 mmol/l; and (5) hypoalphalipoproteinemia (high-density lipoprotein (HDL) level <1.04 mmol/l).

### Statistical analysis

Statistical analyses were performed using R statistical software 3.0.1 (The R Foundation for Statistical Computing, Vienna, Austria) and Stata statistical software (version 5.0, Stata Corporation, College Station, TX). Normally distributed quantitative variables were expressed as the mean ± standard deviation. Non-normally distributed quantitative variables were expressed as median values (interquartile range range, 25–75%).The Student’s t test and Rank sum test were used to evaluate differences in general characteristics and laboratory test between participants with and without HBV infection. Categorical variables were expressed as counts and proportions and evaluated using the chi-squared test. Multivariate logistic regression analysis was used to investigate differences in the likelihood of MetS (and each of the five components of MetS) between the HBV positive and negative group. As age, location, ALT level, and fatty liver are known potential confounding factors of the association between HBV and MetS, logistic regression controlling for these factors were also performed. The strength of the association between HBV and MetS was reported as an adjusted odds ratio OR (controlling for age, sex, location, ALT and fatty liver), with the corresponding 95% confidence interval (CI) of the OR and P-value calculated. A P-value < 0.05 was considered statistically significant.

### Methods statement

All methods were carried out in accordance with relevant guidelines and regulations.
